# Genetic Diversity and Phylogenetic Relationships of Mosquitoes (Diptera: Culicidae) in Central Nepal

**DOI:** 10.1002/ece3.73249

**Published:** 2026-03-23

**Authors:** Punya Ram Sukupayo, Deegendra Khadka, Jyoti Maharjan, Ram Chandra Poudel, Tirth Raj Ghimire

**Affiliations:** ^1^ Department of Zoology, Bhaktapur Multiple Campus Tribhuvan University Bhaktapur Nepal; ^2^ Central Department of Zoology Tribhuvan University Kathmandu Nepal; ^3^ Molecular Biotechnology Unit, Nepal Academy of Science and Technology (NAST) Lalitpur Nepal; ^4^ Department of Zoology, Tri‐Chandra Multiple Campus Tribhuvan University Kathmandu Nepal

**Keywords:** *COI* gene, DNA barcoding, genetic diversity, mosquitoes, phylogeny, vector‐borne diseases

## Abstract

Mosquitoes (family: Culicidae) include several species that act as vectors of major human diseases by transmitting pathogens such as *Plasmodium* spp. (malaria), dengue virus (dengue), filarial worms (filariasis), and various other arboviruses. Species from the subfamilies Anophelinae and Culicinae are key transmitters of pathogens. This research aimed to assess genetic diversity and phylogenetic relationships among different species of mosquitoes collected from five districts of central Nepal. The mitochondrial cytochrome c oxidase I (*COI*) and Internal Transcribed Spacer 2 (*ITS2*) genes were employed for genetic diversity. A total of 7223 mosquitoes were gathered, encompassing 18 species from eight genera across an altitudinal range from 62 to 3840 m above sea level. From the collected mosquitoes, 114 high‐quality sequences were obtained. *Ae*. *aegypti* exhibited lower diversity, with a haplotype diversity (Hd) of 0.46 ± 0.20 and a nucleotide diversity (*π*) of 0.006. The significantly negative Tajima's *D* value (−1.76, *p* < 0.05) suggests the possibility of a recent population expansion or the effect of purifying selection on this species. Phylogenetic analysis revealed distinct clades for most genera, while *Aedes* exhibited paraphyly. Intraspecific genetic divergence (K2P distances) was below 2.39%, while interspecific divergence exceeded 8%. The highest interspecific divergence was observed between *Tx*. *splendens* and *An*. *subpictus* (25.28%), and the lowest between *Cx*. *pipiens* and *Cx*. *quinquefasciatus* (0.43%). Haplotype network analysis revealed that *Ae. aegypti* exhibited a star‐like pattern with a widely shared central haplotype and low‐frequency variants, whereas *Ae. albopictus* showed a more structured network with two main clusters. These findings provide valuable information on the genetic diversity and evolutionary relationships among mosquito species in Nepal.

## Graphical abstract

This study presents a molecular survey of mosquitoes in central Nepal, analyzing 18 species across eight genera using mitochondrial *COI* sequencing. The results revealed significant genetic divergence, clarify phylogenetic relationships, and confirm the presence of newly recorded species, including *Aedes japonicus* , *Anopheles farauti* , and *Culex sasai* . These findings provide a foundational genetic dataset to support accurate species identification, biodiversity assessment, and future vector control strategies in Nepal.

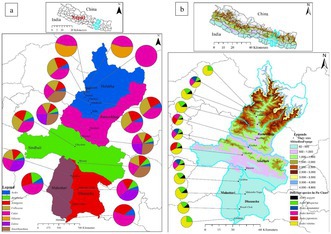

## 1 Introduction

Mosquitoes are dipteran flies, classified under the Family Culicidae Meigen, 1818 [Taxonomic Serial No.: 125930]. They are the most common arthropod vectors responsible for transmitting human diseases. This Family is divided into three Subfamilies: Anophelinae Grassi, 1900 [TSN: 125955]; Culicinae Meigen, 1818 [TSN: 126087]; and Toxorhynchitinae Lahille, 1904 [TSN: 125931]. The Subfamily Toxorhynchitinae, consisting of only one genus, *Toxorhynchites*, is not medically significant because its members feed on nectar rather than blood (Collins and Blackwell 2000; Sukupayo et al. 2024b). In contrast, mosquitoes from the Culicinae, such as *Aedes* and *Culex* (Molina‐Cruz et al. 2013), and those from the Anophelinae Subfamily, like *Anopheles* spp., are important carriers of human pathogens (Sinden 2007).

The Culicidae Family contains 113 genera and 3726 known species, with many more likely yet to be discovered (Harbach 2024). They inhabit a wide range of aquatic and terrestrial environments and have evolved diverse morphological and behavioral adaptations to survive in these settings (Becker et al. 2010). Several mosquito species are of particular concern in tropical medicine and public health because of their transmission of pathogens including nematodes, protozoa, and arboviruses, which cause serious diseases in humans (Killick‐Kendrick 1996). Mosquito‐borne diseases pose major public health challenges, particularly in tropical and temperate zones around the world (Rocklöv and Dubrow 2020). Different diseases transmitted by animal vectors are responsible for more than 17% of all infectious illnesses in humans and cause approximately 700,000 deaths annually (WHO 2020a). Diseases transmitted by mosquitoes, such as malaria, filariasis, dengue, and other arboviral diseases, are of major concerns due to their global impact on public health worldwide (Cook et al. 2005). Consequently, mosquitoes are considered the most significant arthropods, responsible for nearly 90% of major vector‐borne diseases worldwide (WHO 2020a). The increased movement of humans and animals has facilitated the spread and establishment of mosquito vector species, posing significant public health risks worldwide (Anoopkumar et al. 2019). Additionally, mosquitoes are nuisance biters of humans and livestock, which overburden healthcare resources and reduce productivity (Foster and Walker 2019).

Each mosquito species has unique biological and ecological characteristics that influence its potential in disease transmission (Sriwichai et al. 2016; Chaiphongpachara and Laojun 2020). Accurate and timely species identification is essential for understanding disease transmission dynamics and developing effective vector control programs (Sriwichai et al. 2016; Aung et al. 2023). Traditionally, mosquito identification relies on morphological examination, which is time‐consuming, requires specialized expertise, and can be challenging when dealing with damaged specimens or species that are morphologically similar (Bortolus 2008; Batovska et al. 2016).

Dengue fever has become an increasing public health concern in Nepal, with transmission primarily driven by *Aedes* mosquitoes (Mutua et al. 2025; Thakur et al. 2024), particularly members of the subgenus *Stegomyia*. Accurate identification and molecular characterization of these vector species are essential for surveillance and control efforts in regions where morphologically similar species co‐occur. Despite the epidemiological importance of dengue vectors, molecular data on *Aedes* mosquitoes in Nepal remain limited.

To overcome the limitations of traditional morphological methods in mosquito identification, molecular approaches based on mitochondrial DNA sequence variation have been widely incorporated as complementary tools, particularly using regions such as cytochrome c oxidase subunit I (*COI*) (Chaiphongpachara et al. 2022; Hebert et al. 2003). Mitochondrial markers have long been applied in taxonomic and population‐level studies to support morphological identification and to examine genetic variation within and among taxa (Hebert et al. 2003). Such approaches facilitate the processing of large sample sizes and improve consistency in molecular identification, including applications where taxonomic expertise may be limited (Panda and Barik 2020).

The mitochondrial *COI* gene remains a widely used marker in mosquito molecular research due to its maternal inheritance, lack of introns, and comparatively conserved amplification properties (Beebe 2018). Beside *COI* gene, the nuclear Internal Transcribed Spacer 2 (*ITS2*) has also been explored as complementary markers (Beebe 2018). In the present study, mosquitoes collected in Nepal were molecularly characterized primarily using mitochondrial *COI* sequences, with nuclear *ITS2* also amplified as a complementary marker, alongside morphological identification. The study aims to assess genetic variation and phylogenetic relationships within this medically important genus, providing baseline molecular data to support future surveillance and control strategies in Nepal.

## 2 Materials and Methods

### 2.1 Study Area

**FIGURE 1:**
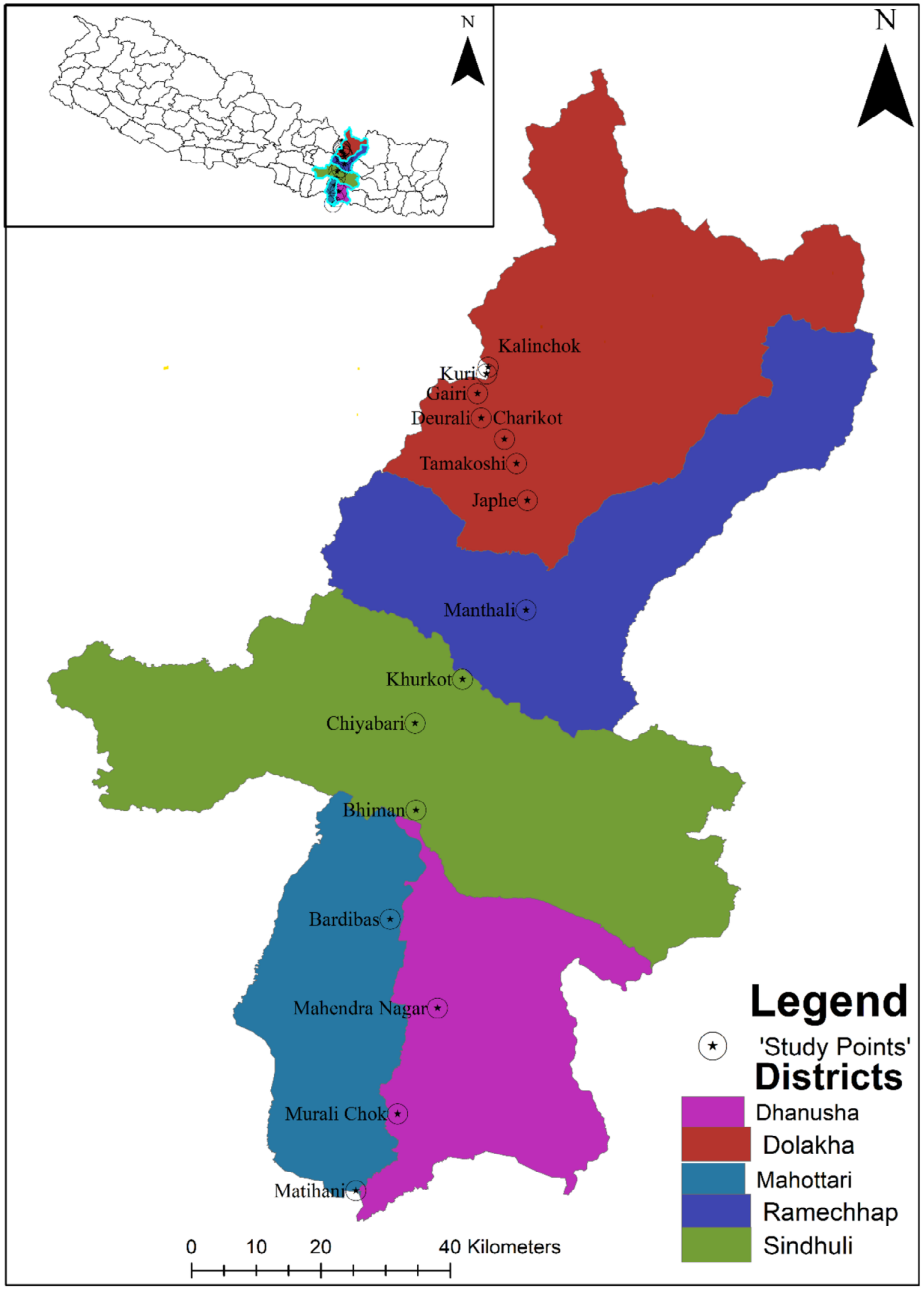
Map of mosquito survey sites in Central Nepal showing sampling locations ranging from Matihani (62 m asl) to Kalinchok (3840 m asl). Each sampling site was georeferenced and assigned a unique identification number using a portable GPS device (etrex 10, GARMIN).

The research was carried out across an altitudinal range from Matihani (26°36′37.56″ N; 85°50′59.54″ E, 62 m asl) of Mahottari district in the south to Kalinchok (27°45′26.59″ N; 86°2′2.66″ E, 3840 m asl) of Dolakha district in the north (Figure 1, Table S1). The study area covers the Mahottari and Dhanusha districts of Madhesh province and the Sindhuli, Ramechhap, and Dolakha districts of Bagmati province. They represent all three geographic regions (Terai, Hill, and Mountain) and all five physiographic regions of Nepal. The five physiographic regions of Nepal are Lowland Terai (< 330 m asl), Lowland Siwalik/Churia (331–700 m asl), Mid Hills (701–2500 m), High Hills (2501–3500 m) and High Mountain (> 3500 m) (Carson et al. 1986). Mahottari and Dhanusha represent the Lowland Terai, Sindhuli spans both Siwalik and Mid Hill regions, Ramechhap represents the Mid Hill, and Dolakha encompasses the High Hill and the High Mountain region. Mosquito surveillance was done at 15 sites following the highway and motorable road from Matihani to Kalinchok, which is the main route followed by both national and international tourists and pilgrims visiting Kuri village and Kalinchok temple located at 3515 and 3840 m respectively (Figure 1).

### 2.2 Entomological Survey

Entomological surveys were conducted from May 2022 to October 2023 to sample both adult and immature (aquatic) stages of container‐breeding mosquitoes, with a primary focus on dengue vectors, particularly *Aedes* species. However, all mosquito taxa encountered during sampling were collected and recorded to document overall mosquito diversity and distribution across different ecological zones. The field surveys were conducted three times each year, in May, July/August, and October, to record seasonal variations in mosquito populations. A door‐to‐door visit was made within a 500‐m radius surrounding each study site. The primary sampling unit comprises private, public, or government properties/residences and their premises. House/premises owner's/occupant's consent was obtained before visiting and collecting the sample from each sampling unit. The properties were divided into eight categories based on whether they had residential quarters, a place of employment, or other public places and meeting areas. All accessible water‐holding containers in all sampling units were examined for immature forms of *Aedes* spp. During the survey, any container with water was defined as a wet container. A container was considered *Aedes* positive if it contained one or more immature *Aedes* mosquitoes. Pipetting (5 mL plastic pipette) or dipping (200 mL dipper) was done to collect samples from positive wet containers. Sampling was taken place at each positive container for around 30 min between 7 a.m. and 6 p.m. The number of dips or pipettes used per habitat varied between 10 and 20, depending on the size of the water container and the amount of water it contained. The larvae or pupae collected were transferred into a plastic bottle along with some water from their original container. The sample bottle was marked with a distinct identification code, along with the collection date, site, and total number of larvae or pupae collected. All breeding habitats associated with all collected mosquitoes were systematically documented using a pre‐design data sheet.

Adult mosquitoes were captured by BG‐Mosquitaire trap (SN: 00101532, Biogents, Germany), baited with BG‐Sweetscent. Following the manufacturer's recommendations, traps were placed in shaded, humid, and wind‐protected outdoor locations near human dwellings—such as within dense vegetation or near water containers—where *Aedes* mosquitoes typically rest and breed. At each study site, traps were operated continuously for 24 h during both pre‐monsoon and post‐monsoon survey periods. Light traps were also employed at night to collect adult mosquitoes.

### 2.3 Morphological Identification

Morphological identification of adult mosquitoes, both those that emerged from collected larvae and pupae and those collected through traps, was conducted using taxonomic keys (Tanaka et al. 1979; Darsie and Pradhan 1990; Schmidt et al. 2001; Darsie 2002; Ree 2003; Huang 2004; Rueda 2004; Darsie and Ward 2005; Rattanarithikul et al. 2005; WHO 2020b). Following species identification based on morphology, mosquito specimens were preserved as vouchered samples and deposited in the museum of the Central Department of Zoology, Tribhuvan University, Nepal (CDZMTU‐DIPCUL013–CDZMTU‐DIPCUL049). To validate morphological identification, DNA was extracted and amplified for molecular taxonomic analysis. Mosquitoes were preserved in a freezer at −20°C until DNA extraction.

### 2.4 DNA Extraction and Quantification

The entire thorax of selected morphologically identified adult mosquitoes served as the source material for the extraction of DNA. The thorax was removed using sterilized forceps and homogenized with 180 μL of PBS (pH 7) using a micro pestle. The entire thorax was processed to ensure sufficient tissue volume for effective homogenization, as isolating only the legs and wings would make the sample difficult to visualize and handle during the procedure. The extraction of DNA was carried out using a DNeasy Blood & Tissue Kit (QIAGEN, Germany), adhering to the protocol provided by the manufacturer. The resulting total DNA was eluted into 100 μL of AE buffer and kept at −20°C until it was used for PCR amplification. A NanoDrop Spectrophotometer (Thermo Scientific, Singapore) was employed to determine the concentration and quality of DNA obtained. DNA samples with a concentration of 100 ng/μL or higher were diluted at a 1:10 ratio before further analysis.

### 2.5 Polymerase Chain Reaction (PCR): Amplification and Cleanup

After successful extraction of DNA, PCR amplification was performed targeting both the mitochondrial *COI* and nuclear *ITS2* genes (Hernández‐Triana et al. 2017). The *COI* gene was amplified using the following primers: the forward primer 5′‐GGAGGATTTGGAAATTGATTAGTTCC‐3′ and the reverse primer 5′‐CCCGGTAAAATTAAAATATAAACTTC‐3′ (Gao et al. 2021; Weeraratne et al. 2018). The *ITS2* region was amplified using the forward primer 5′‐ATCACTCGGCTCATGGATCG‐3′ and the reverse primer 5′‐ATGCTTAAATTTAGGGGGTAGTC‐3′ (Weeraratne et al. 2018). The amplified *COI* and *ITS2* PCR products were subjected to sequencing following standard protocols.

Polymerase chain reactions were conducted in 10 μL volumes, each containing 5 μL 2× PCR Master Mix (Promega), 0.5 μL of a 10 μM solution for both forward and reverse primers, 0.1 μL magnesium chloride, 0.1 μL bovine serum albumin (BSA) (Sigma Aldrich, Germany), 2.8 μL nuclease‐free water (NFW), and 1 μL template DNA. PCR was performed using the ABI 9902 thermocycler (Applied Biosystems, US) with the following temperature cycling profile: an initial denaturation at 95°C for 5 min, followed by 35 repetitions of denaturation at 94°C for 30 s, annealing at either 51°C (for *COI*) or 55°C (for *ITS2*) for 40 s, and extension at 72°C for 45 s. The process concluded with a final extension at 72°C for 10 min.

To ensure the absence of contamination, each PCR included no‐template controls (NTCs), which contained all reaction components except the DNA template. Successful amplification was verified by fractionating the products on 1.5% agarose gels stained with ethidium bromide in 1× Tris‐Acetate‐EDTA (TAE) buffer, followed by visualization using UV light. Only the validated PCR products proceeded to subsequent analysis.

Unwanted primers and dNTPs were removed from the PCR products using the SAP‐Exo Kit (Jena Bioscience, Germany). This involved combining 5 μL of PCR product with 2 μL of SAP‐Exo (Jena Bioscience, Germany) and incubating the mixture at 37°C for 10 min, followed by 80°C for 10 min, according to the manufacturer's protocol.

### 2.6 Sequence Reactions and Sequencing

Sequence reactions were carried out using a thermal cycler (Applied Bio‐systems, US). Each 10 μL reaction mixture consisted of 0.5 μL BigDye Terminator v.3.1 Ready Reaction (RR) Mix (Applied Biosystems, US), 1.75 μL of a 5× Sequencing Buffer (Applied Biosystems, US), 1.5 μL of either the forward or reverse primer, 0.025 μL BSA (Sigma Aldrich, Germany), 5.225 μL nuclease‐free water, and 1 μL of the DNA template. The PCR cycling parameters for sequencing included an initial 1‐min denaturation at 96°C, followed by 40 cycles of 10 s at 96°C (denaturation), 5 s at 50°C (annealing), and 4 min at 60°C (extension). The resulting products were then subjected to Sanger sequencing using an ABI 3500XL Genetic Analyzer (Applied Biosystems, US) at Nepal Academy of Science and Technology (NAST).

### 2.7 Sequence Alignment and DNA Sequence Analysis

The BioEdit (Hall 1999) software was employed to edit raw sequence data. Ambiguous bases were confirmed through multiple sequencing to prevent uncertainty. Forward and reverse reads were then combined to generate a single consensus sequence using Sequencher 4.1.4 (Gene Codes Corporation, USA). To ensure data quality, samples exhibiting abnormalities such as ambiguous base calls or double peaks in chromatograms, which may indicate nuclear mitochondrial pseudogenes (Leite 2012), were excluded. Trace files were carefully trimmed to remove poor‐quality regions, and sequences failing to meet default quality thresholds set in the software were eliminated from the analysis. The resulting nucleotide sequences underwent a comparison against barcode sequences accessible in the NCBI database via BLAST. Subsequently, high‐quality clean sequences were uploaded to NCBI, where they were assigned unique accession codes.

### 2.8 Phylogenetic Analysis and Genetic Diversity

To determine the evolutionary relationships (phylogeny) among the studied mosquito species, Maximum Likelihood (ML) and Neighbor Joining (NJ) methods were employed. The ML analysis was performed using raxmlGUI 2.0 software with bootstrapping for 1000 replicates to assess nodal support (Edler et al. 2021). The general time reversible model with GTR + G + I parameters was used for the analysis. *Chironomus tentans* (family Chironomidae, suborder Nematocera) and *Toxorhynchites* species (Culicidae) were included as outgroups. *C. tentans* is closely related to mosquitoes but lies outside the Culicidae clade, while *Toxorhynchites* is within Culicidae but outside the *Aedes* clade. Using both outgroups allows proper rooting of the phylogeny while maintaining sufficient genetic divergence from the ingroup. FigTree v.1.4.4 (https://tree.bio.ed.ac.uk/software/Figtree/) was employed for the visualization and editing to enhance the clarity of all resulting phylogenetic trees.

Genetic variation within and among the studied species was assessed using the Kimura 2‐Parameter (K2P) model in MEGA 11 (Tamura et al. 2021). DNA sequence polymorphism was analyzed with DnaSP, version 6 (Rozas et al. 2017) to estimate the number of haplotypes, haplotype diversity, parsimony informative sites, variable sites, and GC content. Population genetic statistics such as nucleotide diversity (*π*), haplotype diversity (Hd), and Tajima's *D* were calculated only within individual species, as these metrics are not meaningful across multiple species.

### 2.9 Haplotype Network Analysis

Haplotype network analysis was performed only for species (*Ae. aegypti* and *Ae. albopictus*) that were represented by sufficient sample sizes and with multiple *COI* haplotypes. *COI* sequences from this study were combined with representative sequences from other geographic regions including neighboring countries retrieved from GenBank, with 3–18 sequences selected per country based on sequence quality and alignment overlap. Median‐joining haplotype networks were constructed in PopART v.1.7 (Leigh and Bryant 2015). In the MJ network circle size reflects haplotype frequency, colors indicate geographic origin, hatch marks denote mutational steps, and small black nodes represent inferred median vectors.

## 3 Results

**FIGURE 2:**
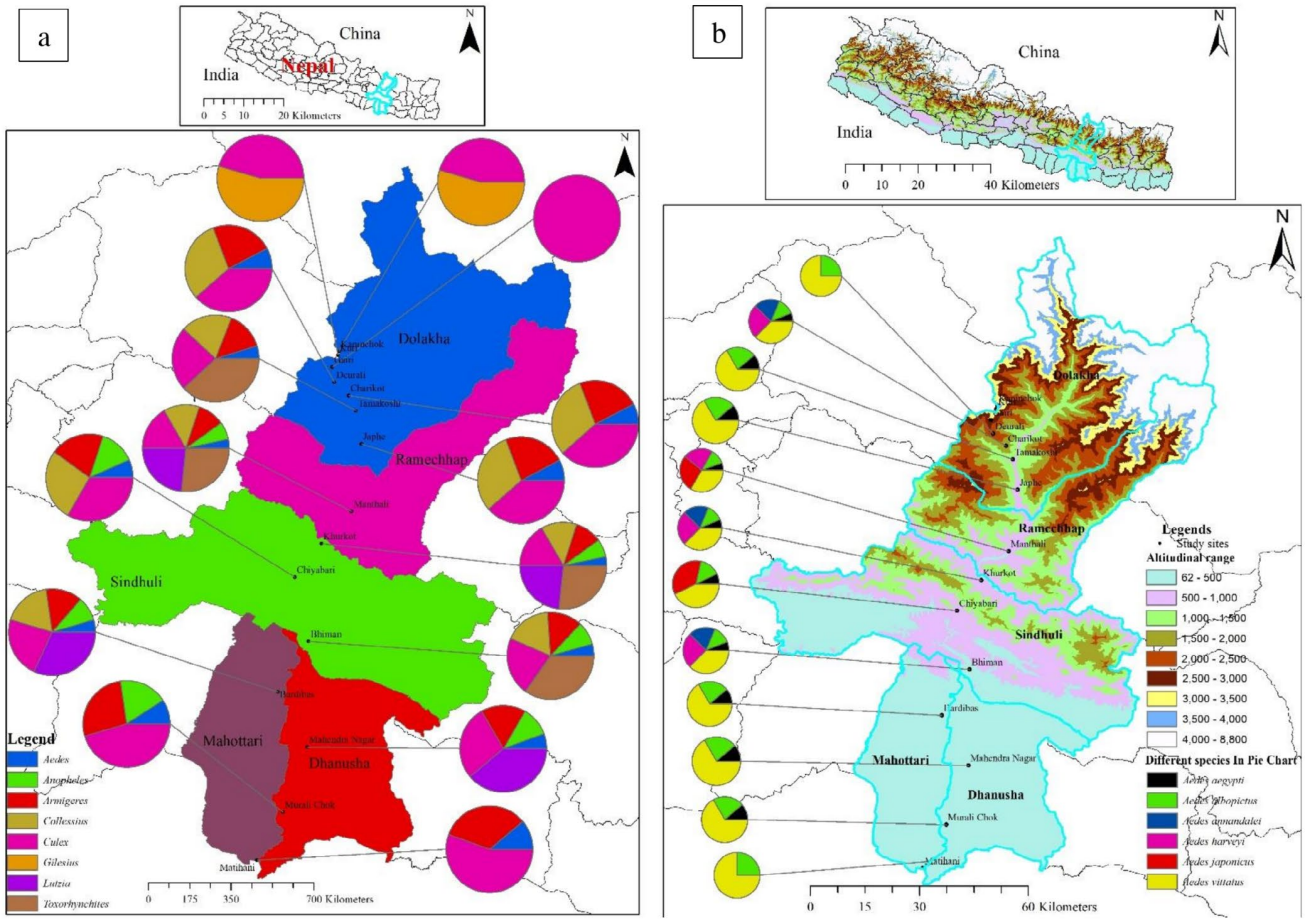
Altitudinal distribution of mosquitoes in Central Nepal. (a) Genus wise distribution. (b) Distribution of *Aedes* mosquitoes.

A total of 7223 mosquito specimens were collected, comprising 3640 immature and 3583 adult individuals. These specimens represented 18 species across 8 genera: *Aedes* (6 species), *Anopheles* (2), *Armigeres* (1), *Collessius* (1), *Culex* (4), *Gilesius* (1), *Lutzia* (1), and *Toxorhynchites* (1). Notably, *Aedes* mosquitoes were found at elevations of up to 2520 m asl, while *Culex*, the most frequently collected genus, was recorded as high as 3840 m asl (Figure 2).

The distribution of mosquito genera varied across districts and altitudinal zones (Figure 2a). *Culex* remains the dominant genus from the southern Terai plains up to the high‐mountain districts of Dolakha. In the lower‐elevation districts of Mahottari and Dhanusha, generic diversity is relatively high, including significant proportions of *Anopheles*, *Aedes*, and *Armigeres*. However, as elevation increases toward the north, generic richness declines, with the northernmost high‐altitude sites in Dolakha exhibiting a simplified community dominated by *Culex* and *Collessius*.

Focusing on the genus *Aedes* (Figure 2b), there is a clear species turnover along the altitudinal gradient from 62 m to over 2500 m. *Ae. albopictus* and *Ae. vittatus* were distributed throughout much of the altitudinal range, while *Ae. aegypti* was largely restricted to lowland urban and semi‐urban areas in the south and recorded over 1900 m. In these lowland areas, the primary disease vectors *Ae. aegypti* and *Ae. albopictus* were most prevalent, while higher elevations show a greater relative abundance of species like *Ae. vittatus*.

### 3.1 DNA Sequence Analysis

The mosquito samples were selected to represent diverse altitudes and ecological conditions to capture a broad range of genetic diversity within each species. Although *ITS2* sequences were initially generated, the majority of the sequences were of low quality for reliable analysis. Consequently, genetic diversity and phylogenetic results are based solely on mitochondrial *COI* sequences. High quality *COI* sequences were successfully obtained from mosquito samples and used for all downstream analysis. A total of 114 *COI* sequences were amplified, representing 18 mosquito species across 8 genera and 3 subfamilies, collected from 15 study sites in central Nepal. The final *COI* sequences were 472 bp long, except for *An. peditaeniatus*, which was 439 bp. All sequences exhibited > 97% identity with the NCBI database entries except for *Aedes japonicus* (95.81%) and *Anopheles farauti* (92.16%). The DNA sequences have been deposited in GenBank and are available under the assigned accession numbers (Table S1).

### 3.2 Nucleotide Composition and Genetic Diversity

Analysis of the nucleotide sequences showed a notable prevalence of adenine and thymine, with the overall AT content averaging 66.73%. The nucleotide frequencies were: T (39.03%), A (27.71%), C (18.27%), and G (15.00%), revealing thymine as the most frequent base and guanine the least. DNA polymorphism analysis revealed 259 invariable sites (52.32%) and 180 variable sites (36.36%), including 172 parsimony informative sites (34.74%) and 8 singleton variable sites (1.62%).

### 3.3 Genetic Diversity Analysis

Sequence alignment revealed 49 variable nucleotide sites, resulting in 37 distinct *COI* sequence variants among the collected mosquito samples. Genetic diversity indices were calculated for *Aedes* species with sufficient sample sizes and multiple haplotypes, while species represented by a single haplotype (*Ae. annandalei*, *Ae. japonicus*, and *Ae. vittatus*) were excluded from population genetic analyses. In *Ae. aegypti*, 11 polymorphic sites were detected, all singleton mutations, and three *COI* haplotypes were observed, resulting in low haplotype diversity (Hd = 0.46 ± 0.20) and low nucleotide diversity (*π* = 0.006). Tajima's *D* was significantly negative (−1.76, *p* < 0.05). *Ae. albopictus* exhibited six polymorphic sites (three singleton and three parsimony‐informative), with six haplotypes, moderate haplotype diversity (Hd = 0.68 ± 0.12), and low nucleotide diversity (*π* = 0.004); Tajima's *D* was negative but not significant (−0.13, *p* > 0.10). In *Ae. harveyi*, 12 polymorphic sites were detected (11 singleton, one parsimony‐informative), with three haplotypes, relatively high haplotype diversity (Hd = 0.83 ± 0.22), and moderate nucleotide diversity (*π* = 0.013); Tajima's *D* was negative but not significant (−0.58, *p* > 0.10) (Table 1).

**TABLE 1:** Genetic diversity and polymorphism statistics of mosquito species based on haplotype and nucleotide variability.

SN	Species	Haplotype no.	Haplotype diversity (Hd)	Nucleotide diversity (*π*)	Variable sites	Singleton variable sites	Parsimony informative sites	Tajima's *D*
1	*Aedes aegypti*	3	0.46 ± 0.20	0.006	11	11	0	−1.76^a^
2	*Ae. albopictus*	6	0.68 ± 0.12	0.004	6	3	3	−0.13
4	*Ae. harveyi*	3	0.83 ± 0.22	0.013	12	11	1	−0.58
9	*An. subpictus*	2	0.50 ± 0.27	0.001	1	1	0	−0.61
11	*Collessius pseudotaeniatus*	8	0.84 ± 0.05	0.011	13	1	12	1.51
16	*Gilesius pulchriventer*	3	0.68 ± 0.12	0.007	6	0	6	2.03^a^
20	For genus *Aedes*	15	0.90 ± 0.03	0.099	132	28	104	0.88
21	For genus *Anopheles*	4	0.79 ± 0.11	0.094	87	61	24	0.95
22	For genus *Culex*	4	0.65 ± 0.12	0.051	66	53	13	0.45

^a^ Suggests deviations from neutrality.

### 3.4 *Anopheles* and *Culex* Species

To provide a broader overview of genetic variation within additional mosquito genera, diversity indices were calculated at the genus level for *Anopheles* and *Culex*, representing interspecific *COI* sequence variation rather than population‐level diversity. In *Anopheles*, 87 variable sites and four haplotypes were detected, whereas *Culex* showed 66 variable sites and four haplotypes. *Anopheles* exhibited high haplotype diversity (Hd = 0.79 ± 0.11), while *Culex* showed moderate haplotype diversity (0.65 ± 0.12). Nucleotide diversity was higher in *Anopheles* (*π* = 0.094) than in *Culex* (*π* = 0.051). Tajima's *D* values were positive but not statistically significant for both genera (Table 1).

### 3.5 Phylogenetic Analysis

**FIGURE 3:**
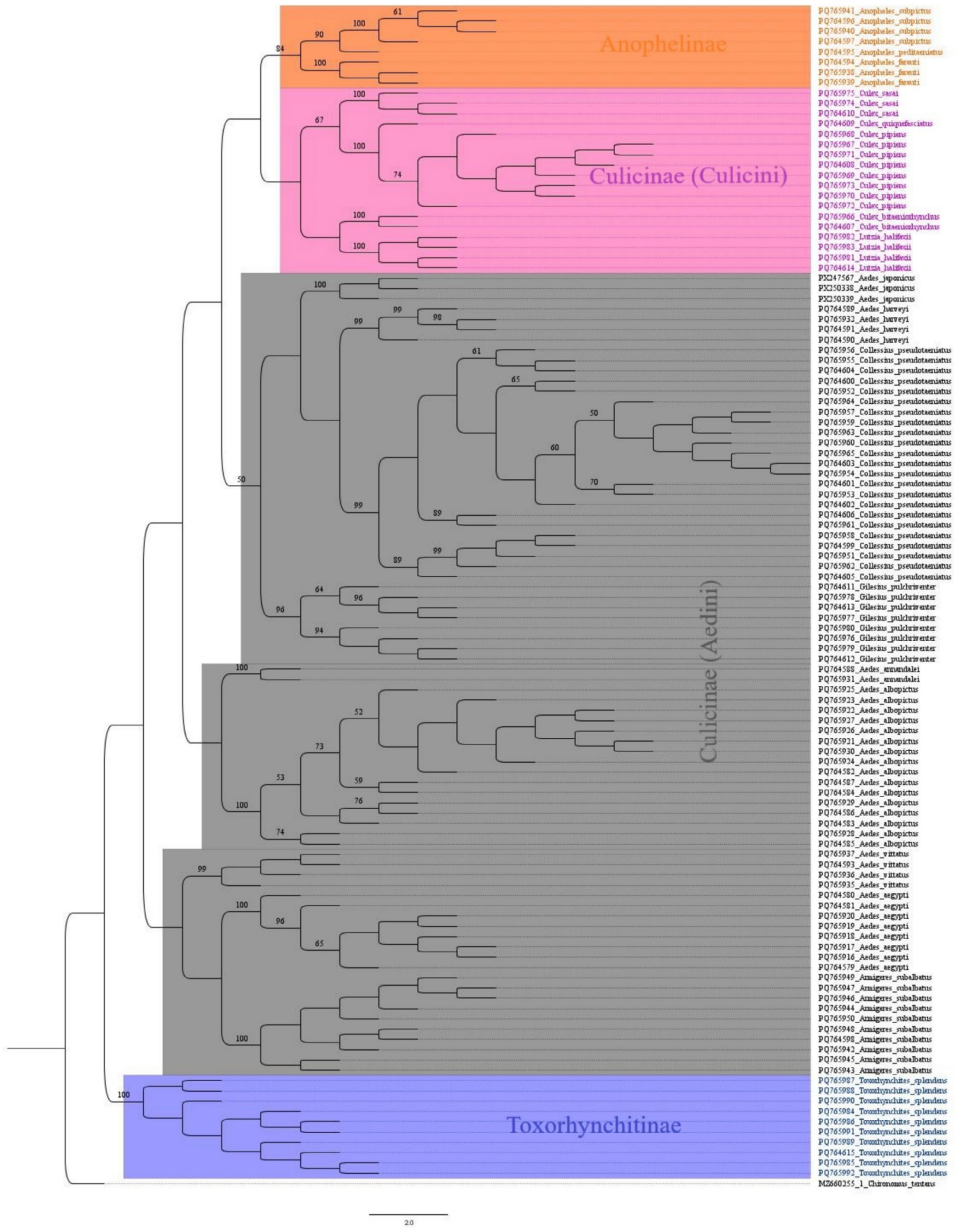
Maximum likelihood phylogenetic tree of the mitochondrial cytochrome oxidase subunit I (*COI*) sequences of mosquitoes with *Chironomus tentans* as outgroup.

The phylogenetic tree effectively resolved all species into distinct monophyletic clades with strong bootstrap support. While most genera (*Anopheles*, *Armigeres*, *Collessius*, *Culex*, *Gilesius*, *Lutzia*, and *Toxorhynchites*) formed monophyletic clades, *Aedes* demonstrated paraphyletic. Within *Aedes*, *Ae*. *annandalei* and *Ae*. *albopictus* formed a distinct clade, while other species clustered with members of different genera: *Ae*. *harveyi* with *Collessius pseudotaeniatus*, *Ae*. *japonicus* with *Gilesius pulchriventer*, *Ae*. *vittatus* with *Armigeres subalbatus*, and *Ae*. *aegypti* formed a separate clade. Similarly, within the *Culex* genus, *Cx*. *pipiens*, *Cx*. *sasai*, and *Cx. quinquefasciatus* formed a well‐supported monophyletic clade, while *Cx*. *bitaeniorhynchus* clustered with *Lutzia halifaxii* (Figure 3).

**FIGURE 4:**
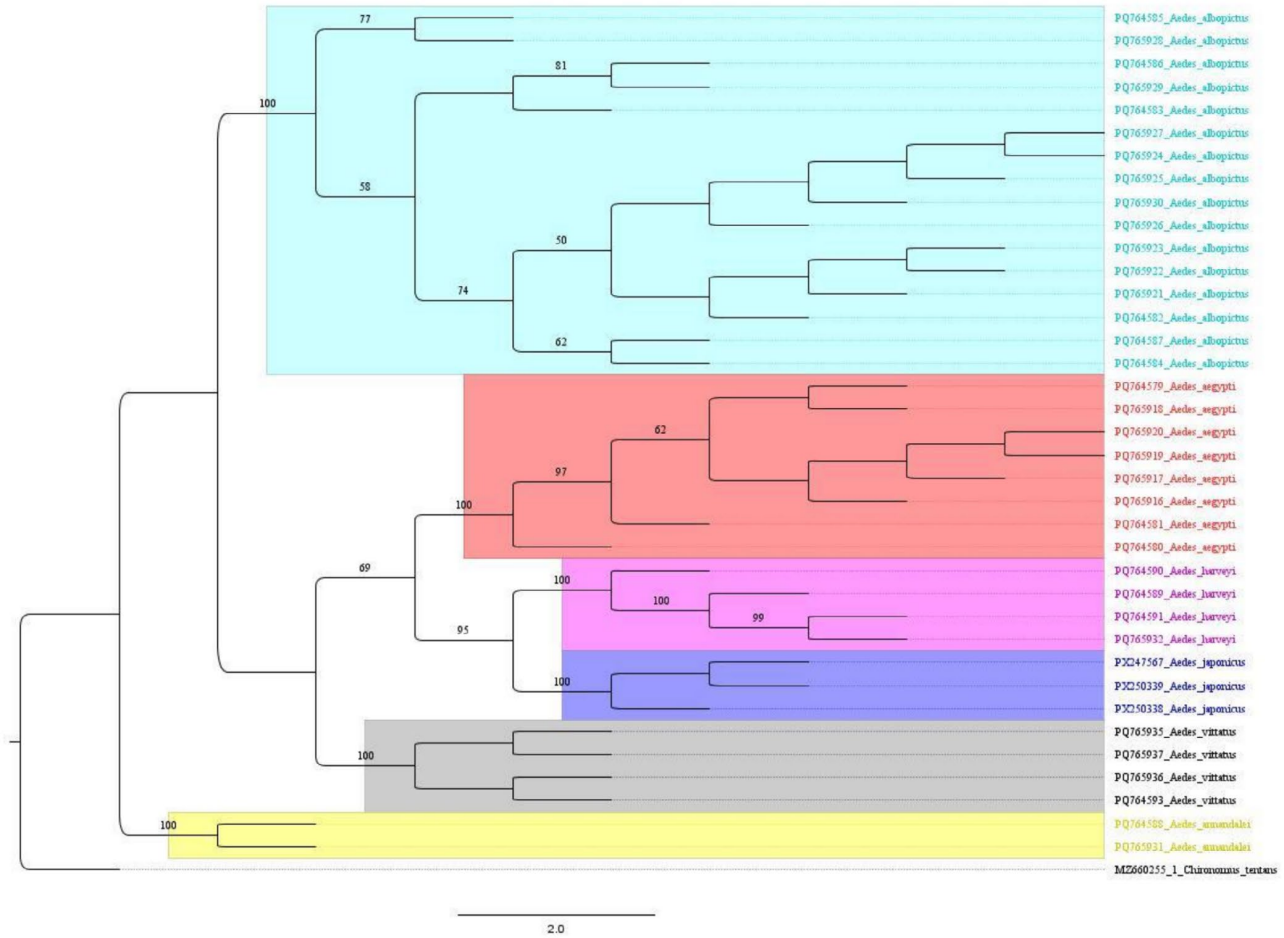
Maximum likelihood phylogenetic tree of the mitochondrial cytochrome oxidase subunit I (*COI*) sequences of *Aedes* mosquitoes with *Chironomus tentans* as outgroup.

Phylogenetic analysis of the genus *Aedes* revealed distinct clusters for several species, including *Ae*. *albopictus*, *Ae*. *aegypti*, *Ae*. *vittatus*, *Ae*. *harveyi*, *Ae*. *japonicus*, and *Ae*. *annandalei*, with high bootstrap support indicating robust evolutionary relationships. *Ae*. *albopictus* exhibited significant intraspecific genetic diversity, likely reflecting its adaptive versatility and widespread geographic distribution. Conversely, *Ae*. *aegypti* formed a tightly clustered group, suggesting lower genetic diversity within the sampled population. Other species, such as *Ae*. *japonicus*, *Ae*. *harveyi*, *Ae*. *vittatus*, and *Ae*. *annandalei*, displayed moderate genetic diversity and formed distinct groups, emphasizing their evolutionary divergence. The close relationship between *Ae*. *harveyi* and the newly reported *Ae*. *japonicus* was evident, suggesting shared ancestry (Figure 4).

### 3.6 Genetic Divergence

Genetic divergence was assessed using the K2P distance method. Intraspecific K2P distances were consistently below 2.39%, with an average of 0.83% (range: 0%–2.39%). *Ae*. *aegypti* exhibited the highest average intraspecific divergence at 1.79% (range: 0.21%–2.39%), followed by *Ae*. *harveyi* at 1.77% (range: 0.85%–2.37%). In contrast, interspecific genetic variation consistently exceeded 8% (except for *Cx*. *pipiens* and *Cx*. *quinquefasciatus*) with an average minimum distance to the nearest neighbor of 16.04% (range: 0.43%–25.28%). The greatest interspecific divergence was observed between *Tx*. *splendens* and *An*. *subpictus* (25.28%), while the lowest interspecific divergence was between *Cx*. *pipiens* and *Cx*. *quinquefasciatus* (0.43%) (Table 2).

**TABLE 2:** Pairwise genetic distances based on *COI* sequences.

	*Ae. aeg*	*Ae. alb*	*Ae. ann*	*Ae. har*	*Ae. jap*	*Ae. vit*	*An. far*	*An. ped*	*An. sub*	*Ar. sub*	*Co. pse*	*Cx. bit*	*Cx. pip*	*Cx. qui*	*Cx. sas*	*G. pul*	*L. hal*	*Tx. spl*
*Ae. aeg*	**0.018**																	
*Ae. alb*	0.143	**0.006**																
*Ae. ann*	0.155	0.109	**0.000**															
*Ae. har*	0.146	0.161	0.163	**0.018**														
*Ae. jap*	0.166	0.176	0.171	0.150	**0.000**													
*Ae. vit*	0.132	0.127	0.119	0.127	0.139	**0.000**												
*An. far*	0.193	0.178	0.192	0.184	0.192	0.169	**0.000**											
*An. ped*	0.200	0.174	0.179	0.185	0.179	0.162	0.154	**0.000**										
*An. sub*	0.182	0.179	0.198	0.188	0.190	0.166	0.162	0.126	**0.002**									
*Ar. Sub*	0.156	0.152	0.163	0.148	0.187	0.124	0.213	0.194	0.199	**0.000**								
*Co. pse*	0.174	0.152	0.147	0.142	0.155	0.173	0.178	0.160	0.201	0.217	**0.012**							
*Cx. bit*	0.139	0.145	0.137	0.145	0.140	0.129	0.170	0.145	0.166	0.165	0.137	**0.000**						
*Cx. pip*	0.122	0.140	0.137	0.150	0.179	0.134	0.172	0.162	0.166	0.153	0.165	0.090	**0.000**					
*Cx. qui*	0.127	0.140	0.137	0.150	0.179	0.134	0.170	0.160	0.166	0.150	0.171	0.090	0.004	**0.000**				
*Cx. sas*	0.132	0.132	0.129	0.150	0.163	0.142	0.179	0.180	0.188	0.155	0.182	0.120	0.097	0.095	**0.000**			
*G. pul*	0.161	0.158	0.121	0.135	0.127	0.107	0.181	0.159	0.190	0.187	0.142	0.124	0.142	0.142	0.142	**0.00964**		
*L. hal*	0.163	0.134	0.137	0.145	0.142	0.144	0.158	0.137	0.165	0.168	0.142	0.088	0.097	0.097	0.117	0.147	**0.000**	
*Tx. spl*	0.227	0.217	0.214	0.204	0.237	0.184	0.233	0.221	0.253	0.209	0.198	0.198	0.209	0.209	0.226	0.240	0.192	**0.000**

*Note:* Intra‐specific average distances are bolded on the diagonal, while inter‐specific distances are presented below the diagonal.

### 3.7 Molecular Characterization and Phylogenetic Relationships of New Country Records

Morphologically identified new country records (*Ae. japonicus*, *An. farauti* and *Cx*. *sasai*) specimens were subjected to *COI* gene amplification and sequencing. The amplified fragments, of 472 base pairs in size, were deposited in GenBank and assigned accession numbers (Table S1).

**FIGURE 5:**
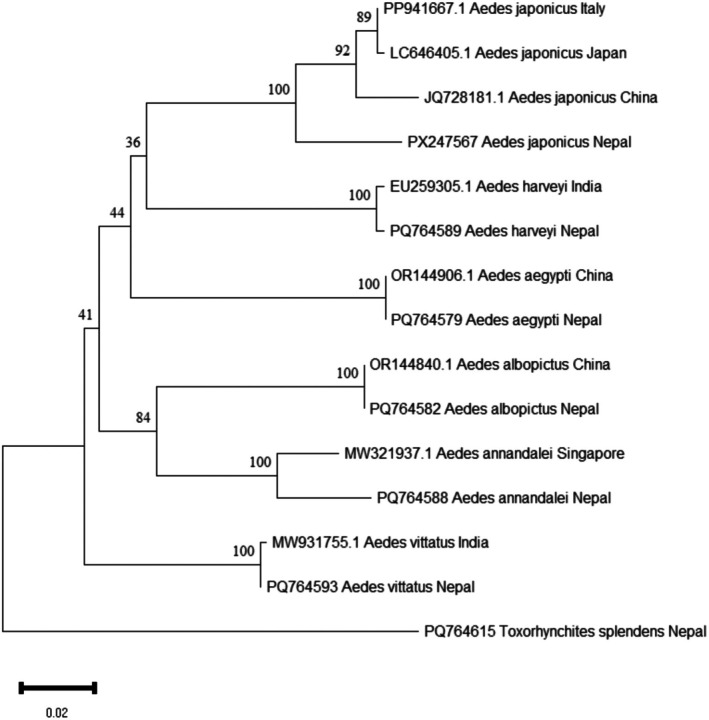
Neighbor‐Joining phylogenetic tree of the mitochondrial *COI* sequences of *Aedes* mosquitoes with *Tx. splendens* as outgroup.

**FIGURE 6:**
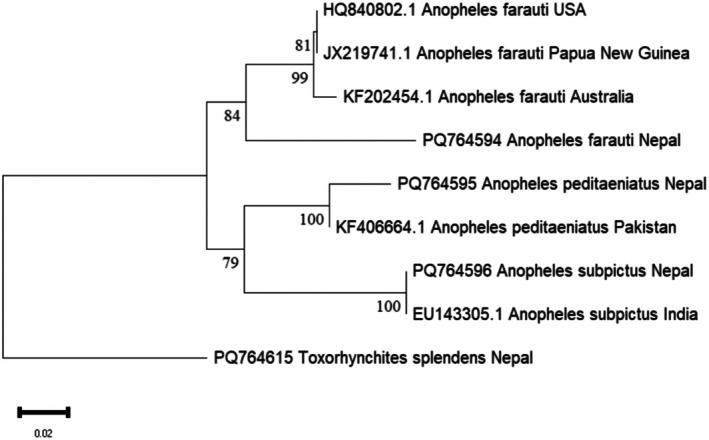
Neighbor‐Joining phylogenetic tree of the mitochondrial *COI* sequences of *Anopheles* mosquitoes with *Tx. splendens* as outgroup.

BLASTN analysis of the *COI* sequences revealed that *Cx. sasai* shared 100% similarity with GenBank entries such as OP927043.1 (China) and KY856956.1 (Bhutan), confirming species identity. *Ae. japonicus* sequences showed a maximum similarity of 96.13% with GenBank entries from Italy such as PP941667.1 and ON911330.1, while *An. farauti* sequences exhibited 92.16% similarity with reference sequences like JX219741.1 (Papua New Guinea). The *Ae. japonicus* sequences from Nepal clustered strongly within the *Ae. japonicus* clade, which included sequences from different countries like China, Italy, and Japan, with high bootstrap support (100%) (Figure 5). Similarly, *An. farauti* sequences grouped robustly with sequences from Papua New Guinea, USA, and Australia, with strong bootstrap support (84%) (Figure 6).

### 3.8 Haplotype Network Analysis of *Aedes aegypti* and *Aedes albopictus*

**FIGURE 7:**
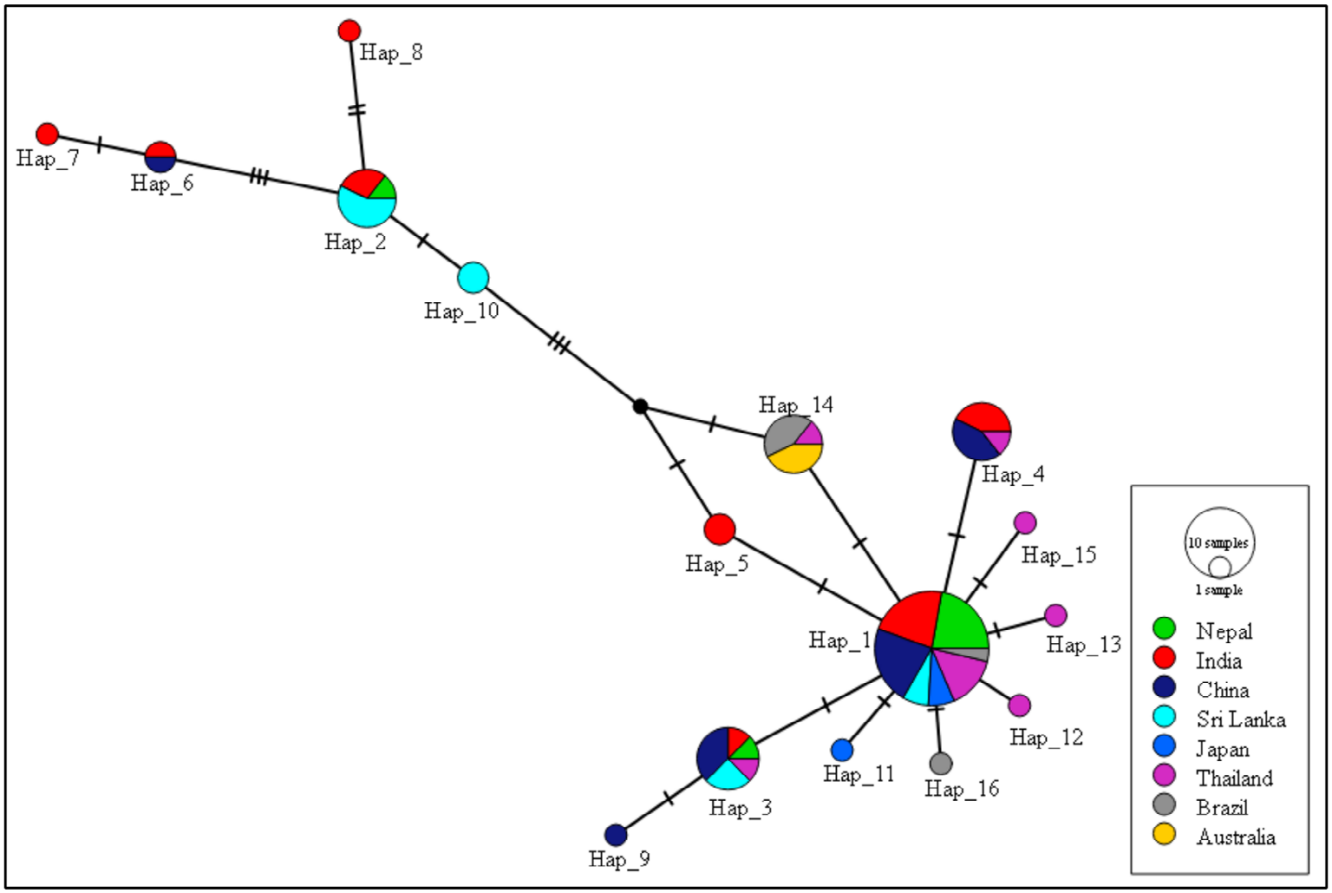
Haplotype network of *Aedes aegypti*.

In *Ae. aegypti*, multiple haplotypes were identified, with one dominant haplotype (Hap_1) occupying a central position in the network. This haplotype was widely shared among populations from Nepal, India, China, Sri Lanka, Japan, Thailand, Brazil, and Australia, and showed a star‐like pattern with several low‐frequency haplotypes radiating from it. Several haplotypes were unique to specific regions, including Nepal, and were separated from the central haplotype by one or a few mutational steps, indicating recent diversification (Figure 7).

**FIGURE 8:**
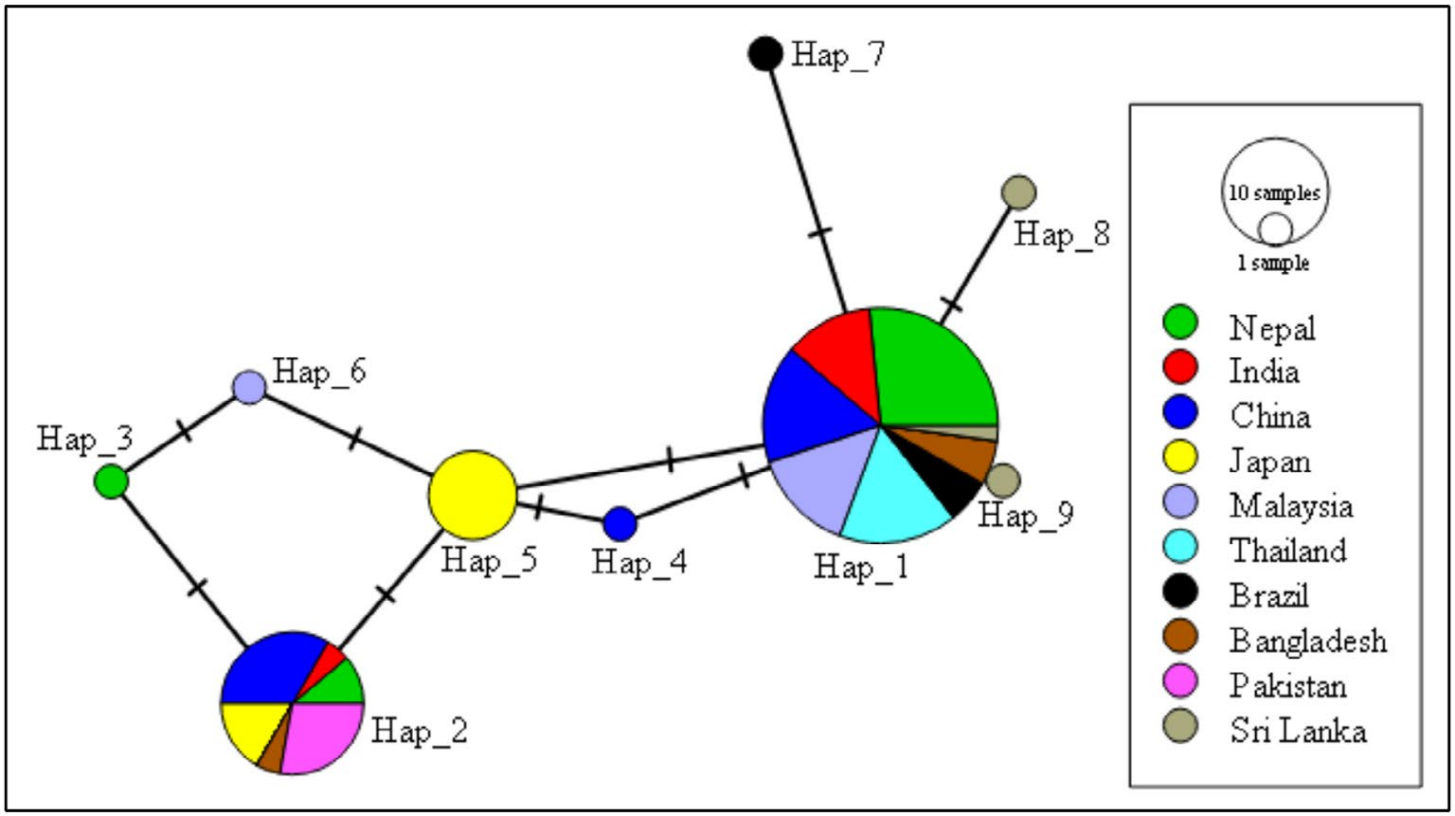
Haplotype network of *Aedes albopictus*.

In *Ae. albopictus*, the haplotype network revealed a moderately complex structure with multiple haplotypes forming two main clusters. A centrally located haplotype (Hap_1) was widely distributed across Nepal and other Asian regions, while additional haplotypes were region‐specific and connected by short mutational distances. A secondary haplotype cluster, including Hap_2, showed limited geographic distribution and greater mutational separation from the central haplotype, suggesting lineage differentiation within the species (Figure 8).

## 4 Discussion

This research aimed to create a DNA barcode database and elucidate genetic structure for common mosquito species in central Nepal, with a focus on their altitudinal distribution. Mosquito samples were collected from diverse regions, identified using both morphological and molecular techniques. The altitudinal gradient in Central Nepal acts as an important environmental filter, shaping mosquito communities from high generic diversity in the tropical lowlands to a simplified structure dominated by *Culex* and *Collessius* at higher elevations. *Culex* demonstrates broad ecological tolerance across the gradient, while the genus *Aedes* shows pronounced species turnover. *Ae. aegypti* is largely restricted to lowland urban and semi‐urban areas but has been recorded over 1900 m, indicating some adaptability to higher elevations. In contrast, *Ae. albopictus* and *Ae. vittatus* are distributed more broadly. These patterns suggest that altitudinal and climatic factors strongly influence mosquito community composition, and the observed upward range extension of tropical vectors like *Ae. aegypti* signals a potential increase in arboviral risk for previously unaffected mid‐ and high‐altitude communities.

A total of 114 high quality *COI* sequences, representing 18 species across 8 genera, were analyzed. While mitochondrial *COI* is widely used for mosquito molecular identification, nuclear *ITS2* have also been explored; however, consistent with previous reports, *ITS2* amplification in this study did not yield sequences of sufficient quality for analysis due to the presence of multiple tandemly repeated copies of rRNA genes in the mosquito genome (Beebe 2018; Calzolari et al. 2021; Madeira et al. 2021). The mitochondrial *COI* gene proved highly effective for distinguishing most species, highlighting its utility for mosquito surveillance and research (Hebert et al. 2003; Ashfaq et al. 2014). DNA barcoding complements traditional morphological identification, particularly when specimens are of low quality or when morphological keys are challenging to use (Bortolus 2008; Batovska et al. 2016). Accurate species identification is essential for implementing successful vector control strategies (Sriwichai et al. 2016; Aung et al. 2023). Mitochondrial genes, especially *COI*, are ideal for molecular studies in insects due to their abundance, maternal inheritance, and rapid evolutionary rate compared to nuclear genes. The molecular analysis provided insights into species diversity, phylogeny, and population genetics, all of which are essential for vector surveillance and control programs. The morphological identification of all mosquitoes was supported by phylogenetic analysis using *COI* sequences, highlighting the benefit of combining these two approaches.

The generated *COI* sequences exhibited a high AT‐richness, with nucleotide frequencies of A = 27.71%, T = 39.03%, C = 18.27%, and G = 15.00%, resulting in an average AT‐richness of 66.73%. This finding aligns with previous research (Cywinska et al. 2006; Panda and Barik 2022), highlighting a consistent pattern of AT‐richness across dipteran taxa. Furthermore, these results are consistent with other studies on mosquito *COI* sequences, which reported similar levels of AT‐richness (Chaiphongpachara et al. 2022; Lamichhane et al. 2024). The observed AT‐richness in mosquito *COI* sequences is a characteristic commonly found within this gene region across diverse dipteran taxa. This consistent pattern likely reflects inherent evolutionary pressures and genomic biases that have influenced the nucleotide composition of this important mitochondrial gene.

For specific identification, a 2% to 3% pairwise genetic divergence in *COI* sequences has been widely accepted as a benchmark (Hebert et al. 2003), and this has been applied to closely related mosquito species as well (Chan et al. 2014). Analyzing 114 mosquito *COI* haplotypes revealed significant differences between inter and intraspecific genetic divergence. Notably, interspecific *COI* sequence differences were, on average, nearly 20 times greater than intraspecific differences. Furthermore, interspecific K2P distances exceeded 0.8 (8%) for all mosquito species, confirming them as distinct species. However, the remarkably lower interspecific distance of 0.00426 (0.43%) between *Cx. pipiens* and *Cx. quinquefasciatus* indicated a much closer genetic relationship. The interspecific K2P divergence observed in this study, ranging from 8.76% to 25.28%, aligns with the variability reported in previous barcoding investigations in various countries, including Sri Lanka (7.2%–17.2%; Weeraratne et al. 2017), India (5.87%–25.65%; Kumar et al. 2007), China (2.3%–21.8%; Wang et al. 2012), and Pakistan (2.3%–17.8%; Ashfaq et al. 2014). However, an exception was noted in the case of *Cx. pipiens* and *Cx. quinquefasciatus*, which showed a much lower divergence of 0.43%. The interspecific K2P distance between *Cx. pipiens* and *Cx. quinquefasciatus* was observed to be 0.43%, which is significantly lower than the typical interspecific divergence thresholds (2%–3%) reported in mosquito DNA barcoding studies (Hebert et al. 2003; Kumar et al. 2007). These two species are recognized members of the closely related *Cx. pipiens* complex, characterized by overlapping morphological and ecological characteristics. Hybridization between *Cx. pipiens* and *Cx. quinquefasciatus* has been well‐documented (Bahnck and Fonseca 2006; Gomes et al. 2012), leading to the exchange of genetic material (introgression) (Gomes et al. 2012), which might explain the low divergence observed in the current study.

Understanding the genetic diversity of disease vectors is crucial for accurate species identification and for resolving phylogenetic relationships among closely related taxa. Such information supports targeted surveillance and control strategies by revealing the presence of cryptic species or newly introduced populations that might otherwise remain undetected.

The haplotype network analyses provided insights into the genealogical relationships and global connectivity of *Ae. aegypti* and *Ae. albopictus* populations. In *Ae. aegypti*, the centrally positioned and widely distributed haplotype likely represents a common ancestral or founder lineage that has dispersed across multiple geographic regions. The star‐like topology of the network, together with the predominance of singleton mutations, low genetic diversity (Hd = 0.46 ± 0.20, *π* = 0.006) and a significantly negative Tajima's *D* (−1.76, *p* < 0.05), collectively indicate recent population expansion, potentially facilitated by human‐mediated dispersal and urbanization. This pattern, consistent with large‐scale studies (Escobar et al. 2022; Gloria‐Soria et al. 2016), likely reflects historical bottlenecks associated with colonization events outside Africa, where higher allelic richness supports its origin (Bennett et al. 2016). Reduced genetic variability in *Ae. aegypti* populations outside of Africa may have practical implications for vector control: low genetic diversity could make populations more susceptible to targeted interventions, such as *Wolbachia* releases or gene drive strategies, by limiting their adaptive capacity. It may also reduce the likelihood of rapid resistance development to insecticides or other control measures, particularly in isolated populations. Conversely, areas with high genetic diversity might require more integrated and adaptable control strategies.

In contrast, *Ae. albopictus* exhibited a more complex network structure. Although a central, widely shared haplotype likely represents a founder lineage, the presence of multiple region‐specific haplotypes indicates ongoing diversification and geographic structuring. The secondary cluster, including Hap_2, appears to represent a derived lineage based on its peripheral position and greater mutational distance from the network center. The non‐significant Tajima's *D* value suggests demographic stability or only weak population expansion in *Ae. albopictus* compared to *Ae. aegypti*.

Beyond *Aedes*, genus‐level analysis revealed contrasting patterns of interspecific *COI* variation between *Anopheles* and *Culex*. Higher haplotype and nucleotide diversity in *Anopheles* indicate greater genetic divergence among the included species, suggesting broader evolutionary separation. In contrast, the lower nucleotide diversity in *Culex* implies that the analyzed species are more closely related or exhibit reduced *COI* divergence. Although Tajima's *D* values were positive for both genera, their non‐significance and the interspecific nature of the dataset limit demographic or selective interpretations. Instead, these values likely reflect lineage structure rather than population‐level evolutionary processes.

From a vector control perspective, these findings imply that control programs must consider the genetic variability of these populations, as high genetic diversity may enable rapid evolution of resistance traits. Therefore, integrated vector management approaches combining chemical, biological, and environmental interventions are essential to sustainably manage these disease vectors.

Maximum‐likelihood (ML) trees are widely used in phylogenetic analyses, particularly within mosquito species, to illustrate evolutionary relationships among targeted groups (Chan et al. 2014). The mitochondrial *COI*‐based analysis provided strong support for the placement of all 18 species within distinct monophyletic clusters, confirming their taxonomic assignments. The GTR + G + I model was selected for ML analysis of the 114 sequences representing the partial *COI* gene sequence. A tree topology was automatically computed for estimating ML values, with a final optimization likelihood of −3672.53, indicating a good fit of the model to the data. Notably, *Culex* species formed a well‐defined clade with distinct subclades for *Cx*. *pipiens*, *Cx*. *quinquefasciatus*, *Cx*. *bitaeniorhynchus*, and *Cx*. *sasai*, reflecting their close evolutionary lineage. *Aedes* species exhibited distinct clades, with high bootstrap support emphasizing their genetic divergence. Key vectors like *Ae*. *aegypti* and *Ae*. *albopictus* were clearly separated, highlighting their independent evolutionary histories. Furthermore, the distinct clustering of *Ae*. *vittatus* and *Ae*. *japonicus* further highlighted the evolutionary diversity within the *Aedes* genus. *Anopheles* species, including *An*. *subpictus* and *An*. *farauti*, formed a distinct clade, suggesting moderate genetic variation within this group. Non‐blood‐feeding species, such as *Tx*. *splendens*, formed a distinct lineage, demonstrating their evolutionary divergence from hematophagous genera. Similarly, *Ar*. *subalbatus* and *Gilesius pulchriventer* emerged as unique clades, reflecting their specialized ecological niches and independent evolutionary paths. The results of this study offer significant understanding of evolutionary relationships and the process of diversification within the mosquito family.

Our findings align with previous studies indicating that the genus *Aedes* is not a monophyletic group (Da Silva et al. 2020; Soghigian et al. 2017). Overall, *Aedes* exhibits paraphyly, as demonstrated by multiple phylogenetic inconsistencies across various studies that used different genetic markers and representative species. This highlights the need for further reclassification based on the current phylogenetic understanding of these species.

The three newly reported species were morphologically conformed (Sukupayo et al. 2024a). However, only *Cx. sasai* achieved 100% *COI* sequence similarity with NCBI reference sequences. The *COI* similarities for *Ae. japonicus* (95.81%) and *An. farauti* (92.16%) were lower than the commonly used species‐level similarity range (≈97%–98%) for *COI* barcoding (Hebert et al. 2003). However, species identification was supported by strong phylogenetic clustering with high nodal support within their respective species groups, in agreement with morphological characteristics. As a globally invasive species, *Ae. japonicus* has undergone multiple introductions and exhibits genetic divergence between populations in its introduced range, with evidence of distinct genotypes and haplotype structures (Janssen et al. 2020). Thus, the comparatively lower *COI* similarity in Nepal may reflect substantial intraspecific variation or a distinct regional lineage resulting from geographic isolation and local adaptation.

Similarly, the case of *An. farauti* is particularly noteworthy given its lower *COI* similarity (92.16%). Sequences of *An. farauti* submitted from different regions form single cluster with good nodal support, which is stronger than the nodal support observed for sequences with accession numbers HQ840802 (USA) and JX219741.1 (Papua New Guinea) (Figure 6). This substantial genetic divergence, coupled with the consistent clustering of Nepalese *An. farauti* sequences within the *An. farauti* in the phylogenetic tree, suggests several possibilities. Firstly, it could represent a highly divergent population of a known species within the complex, adapted to the Nepalese environment recently. Secondly, and perhaps more significantly, it might indicate the presence of a previously uncharacterized cryptic species within the *An. farauti* complex that is morphologically identical but genetically distinct. Given the broad distribution of the *An. farauti* complex primarily in the Australasian and Oceanic regions, its establishment in Nepal, along with this unique genetic signature, warrants serious attention. Such divergence highlights the limitations of relying solely on a single barcoding gene for species identification, especially in species complexes where morphological characters are insufficient for differentiation and genetic diversity is high. Further genetic work using additional markers (e.g., *ITS2*, ND4, or whole mitochondrial genome) is suggested to clarify the taxonomic status of these Nepalese *An. farauti* populations.

This study employed mitochondrial *COI* gene sequencing to examine the genetic diversity and evolutionary relationships among 18 mosquito species across eight genera in Nepal. The analysis revealed substantial genetic differences between species, with most genera forming well‐defined evolutionary groups. This research also presents molecular data supporting the existence of species newly reported in Nepal, including *Aedes japonicus*, *Anopheles farauti* , and *Culex sasai*. However, it is limited by the inclusion of a small number of species, which may not fully represent the diversity within each genus. Additionally, the analysis focused on a single gene region, which may not capture the complete genetic diversity of these species. Although *COI* is widely used for species identification and barcoding, it alone cannot fully resolve intraspecific variation, cryptic species, or deeper phylogenetic relationships. Future studies incorporating additional mitochondrial (e.g., ND4, 16S rRNA) and nuclear markers, or genome‐wide approaches, would provide a more comprehensive understanding of mosquito genetic diversity and population structure in Nepal. Incorporating genomic‐scale data, such as whole‐genome sequencing, would lead to a more complete picture of genetic variation and population structure. Furthermore, integrating spatial and temporal data could shed light on the impact of geographic distribution and temporal dynamics on genetic diversity patterns.

## 5 Conclusion

This study provides molecular evidence confirming the distribution of *Aedes japonicus*, *Anopheles farauti* , and *Culex sasai* in Nepal. The comprehensive analysis of 18 mosquito species across eight genera reveals significant genetic divergence, with most genera forming distinct monophyletic clusters. These findings clarify phylogenetic relationships and evolutionary patterns within Nepal's mosquito fauna. The genetic analysis revealed notable intraspecific variation, particularly within *Ae*. *albopictus*, while other species like *Ae*. *aegypti* demonstrated lower diversity, suggesting possible recent population expansion or selection. Interspecific genetic distances were substantial, with some species pairs showing remarkably high divergence, such as *Tx*. *splendens* and *An*. *subpictus*, underscoring the complex evolutionary relationships within the Culicidae family. This research provides crucial genetic data that enhances our understanding of mosquito biodiversity in Nepal. Genetic profiling enables accurate species identification and distribution mapping, even among morphologically similar species. This foundational dataset is essential for future monitoring efforts, allowing researchers to track changes in mosquito populations over time and to evaluate the effectiveness of emerging vector control strategies.

## Author Contributions

**Punya Ram Sukupayo:** conceptualization (equal), data curation (equal), formal analysis (equal), investigation (equal), methodology (equal), project administration (equal), software (equal), writing – original draft (equal), writing – review and editing (equal). **Deegendra Khadka:** supervision (equal), validation (equal), writing – review and editing (equal). **Jyoti Maharjan:** resources (supporting), supervision (equal), validation (equal), visualization (equal), writing – review and editing (equal). **Ram Chandra Poudel:** data curation (equal), methodology (equal), resources (lead), software (equal), supervision (equal), validation (equal), visualization (equal), writing – review and editing (equal). **Tirth Raj Ghimire:** conceptualization (equal), resources (supporting), supervision (equal), validation (equal), visualization (equal), writing – review and editing (equal).

## Funding

The authors have nothing to report.

## Ethics Statement

The ethical approval was obtained from the Institutional Review Committee (IRC) (Ref. No. IRC/IOST 49/079/080). Similarly, written consent was obtained from Golanjor Rural Municipality (2967/2078/079), Kamalamai Municipality (4534/2078/079), Bardiwas Municipality (3493/2078/079), Janakpurdham Sub‐Metropolitan City (11994/078/079), Matihani Municipality (1785/078/079), Manthali Municipality (3831/078/079), Bhimeshwor Municipality (2700/078/079), and Tamakoshi Rural Municipality (2435/2078/079).

## Conflicts of Interest

The authors declare no conflicts of interest.

## Supporting information


**Data S1:** ece373249‐sup‐0001‐supinfo.docx.

## Data Availability

Data supporting the findings of this study are available within the article and from the corresponding authors upon reasonable request.
