# Supplementary material for: Genetic Diversity and Phylogenetic Relationships of Mosquitoes (Diptera: Culicidae) in Central Nepal
Source: Ecol Evol. 2026 Mar 23;16(3):e73249. doi: 10.1002/ece3.73249 (PMC13093738; doi:10.1002/ece3.73249)
Supplement: Supplementary file 1 — Data S1: ece373249‐sup‐0001‐supinfo.docx. [file ECE3-16-e73249-s001.docx]

**Supplementary Table 1.** Genetic diversity and haplotype analysis of mosquito species with voucher and accession numbers

| S. N. | Mosquitoes | Latitude-Longitude | Voucher no. or (Isolate no.) | Accession Number | Haplotype | Haplotype diversity | Nucleotide diversity | Tajima's D | P |
| --- | --- | --- | --- | --- | --- | --- | --- | --- | --- |
| 1 | *Aedes aegypti* | 26.989 N-85.897 E | CDZMTU-DIPCUL014 | PQ764579 | 3 | 0.46±0.20 | 0.006 | -1.76 | <0.05 |
| 2 | *Aedes aegypti* | 27.324 N-85.995 E | CDZMTU-DIPCUL015 | PQ764580 |  |  |  |  |  |
| 3 | *Aedes aegypti* | 27.548 N-86.085 E | CDZMTU-DIPCUL013 | PQ764581 |  |  |  |  |  |
| 4 | *Aedes aegypti* | 26.718 N-85.908 E | (132K) | PQ765916 |  |  |  |  |  |
| 5 | *Aedes aegypti* | 27.103 N-85.966 E | (R65) | PQ765917 |  |  |  |  |  |
| 6 | *Aedes aegypti* | 27.396 N-86.063 E | (R68) | PQ765918 |  |  |  |  |  |
| 7 | *Aedes aegypti* | 27.623 N-86.073 E | (353K) | PQ765919 |  |  |  |  |  |
| 8 | *Aedes aegypti* | 27.657 N-86.056 E | (354K) | PQ765920 |  |  |  |  |  |
| 9 | *Aedes albopictus* | 26.610 N-85.850 E | CDZMTU-DIPCUL020 | PQ764582 | 6 | 0.68±0.12 | 0.004 | -0.13 | >0.05 |
| 10 | *Aedes albopictus* | 27.396 N-86.063 E | CDZMTU-DIPCUL017 | PQ764583 |  |  |  |  |  |
| 11 | *Aedes albopictus* | 27.623 N-86.073 E | CDZMTU-DIPCUL021 | PQ764584 |  |  |  |  |  |
| 12 | *Aedes albopictus* | 26.989 N-85.897 E | CDZMTU-DIPCUL019 | PQ764585 |  |  |  |  |  |
| 13 | *Aedes albopictus* | 27.248 N-85.935 E | CDZMTU-DIPCUL016 | PQ764586 |  |  |  |  |  |
| 14 | *Aedes albopictus* | 27.687 N-86.024 E | CDZMTU-DIPCUL018 | PQ764587 |  |  |  |  |  |
| 15 | *Aedes albopictus* | 26.718 N-85.908 E | (133K) | PQ765921 |  |  |  |  |  |
| 16 | *Aedes albopictus* | 26.865 N-85.963 E | (139K) | PQ765922 |  |  |  |  |  |
| 17 | *Aedes albopictus* | 26.989 N-85.897 E | (163K) | PQ765923 |  |  |  |  |  |
| 18 | *Aedes albopictus* | 27.103 N-85.966 E | (164K) | PQ765924 |  |  |  |  |  |
| 19 | *Aedes albopictus* | 27.248 N-85.935 E | (R21) | PQ765925 |  |  |  |  |  |
| 20 | *Aedes albopictus* | 27.324 N-85.995 E | (R60) | PQ765926 |  |  |  |  |  |
| 21 | *Aedes albopictus* | 27.548 N-86.085 E | (148K) | PQ765927 |  |  |  |  |  |
| 22 | *Aedes albopictus* | 27.396 N-86.063 E | (366K) | PQ765928 |  |  |  |  |  |
| 23 | *Aedes albopictus* | 27.548 N-86.085 E | (367K) | PQ765929 |  |  |  |  |  |
| 24 | *Aedes albopictus* | 27.657 N-86.056 E | (394K) | PQ765930 |  |  |  |  |  |
| 25 | *Aedes annandalei* | 27.324 N-85.995 E | CDZMTU-DIPCUL022 | PQ764588 | 1 | 0.00±0.00 | 0 | n/a | n.a |
| 26 | *Aedes annandalei* | 27.657 N-86.056 E | (167K) | PQ765931 |  |  |  |  |  |
| 27 | *Aedes harveyi* | 27.103 N-85.966 E | CDZMTU-DIPCUL023 | PQ764589 | 3 | 0.83±0.22 | 0.013 | -0.58 | >0.1 |
| 28 | *Aedes harveyi* | 27.657 N-86.056 E | CDZMTU-DIPCUL025 | PQ764590 |  |  |  |  |  |
| 29 | *Aedes harveyi* | 27.396 N-86.063 E | CDZMTU-DIPCUL024 | PQ764591 |  |  |  |  |  |
| 30 | *Aedes harveyi* | 27.324 N-85.995 E | (144K) | PQ765932 |  |  |  |  |  |
| 31 | *Aedes japonicus* | 27.248 N-85.935 E | CDZMTU-DIPCUL026 | PX247567 | 1 | 0.00±0.00 | 0 | n/a | n.a |
| 32 | *Aedes japonicus* | 27.396 N-86.063 E | (R52) | PX250338 |  |  |  |  |  |
| 33 | *Aedes japonicus* | 27.657 N-86.056 E | (147K) | PX250339 |  |  |  |  |  |
| 34 | *Aedes vittatus* | 26.989 N-85.897 E | CDZMTU-DIPCUL027 | PQ764593 | 1 | 0.00±0.00 | 0 | n/a | n.a |
| 35 | *Aedes vittatus* | 26.718 N-85.908 E | (R18) | PQ765935 |  |  |  |  |  |
| 36 | *Aedes vittatus* | 27.396 N-86.063 E | (R25) | PQ765936 |  |  |  |  |  |
| 37 | *Aedes vittatus* | 27.687 N-86.024 E | (372K) | PQ765937 |  |  |  |  |  |
| 38 | *Anopheles farauti* | 27.324 N-85.995 E | CDZMTU-DIPCUL028 | PQ764594 | 1 | 0.00±0.00 | 0 | n/a | n.a |
| 39 | *Anopheles farauti* | 26.865 N-85.963 E | (141K) | PQ765938 |  |  |  |  |  |
| 40 | *Anopheles farauti* | 27.248 N-85.935 E | (R58) | PQ765939 |  |  |  |  |  |
| 41 | *Anopheles peditaeniatus* | 27.248 N-85.935 E | CDZMTU-DIPCUL029 | PQ764595 | 1 | 0.00±0.00 | 0 | n/a | n.a |
| 42 | *Anopheles subpictus* | 26.718 N-85.908 E | CDZMTU-DIPCUL030 | PQ764596 | 2 | 0.5±0.27 | 0.001 | -0.61 | >0.1 |
| 43 | *Anopheles subpictus* | 27.324 N-85.995 E | CDZMTU-DIPCUL031 | PQ764597 |  |  |  |  |  |
| 44 | *Anopheles subpictus* | 26.989 N-85.897 E | (R9) | PQ765940 |  |  |  |  |  |
| 45 | *Anopheles subpictus* | 27.248 N-85.935 E | (R31) | PQ765941 |  |  |  |  |  |
| 46 | *Armigeres subalbatus* | 26.989 N-85.897 E | CDZMTU-DIPCUL032 | PQ764598 | 1 | 0.00±0.00 | 0 | n/a | n.a |
| 47 | *Armigeres subalbatus* | 26.610 N-85.850 E | (134K) | PQ765942 |  |  |  |  |  |
| 48 | *Armigeres subalbatus* | 26.718 N-85.908 E | (135K) | PQ765943 |  |  |  |  |  |
| 49 | *Armigeres subalbatus* | 26.865 N-85.963 E | (149K) | PQ765944 |  |  |  |  |  |
| 50 | *Armigeres subalbatus* | 27.248 N-85.935 E | (R1) | PQ765945 |  |  |  |  |  |
| 51 | *Armigeres subalbatus* | 27.324 N-85.995 E | (138K) | PQ765946 |  |  |  |  |  |
| 52 | *Armigeres subalbatus* | 27.396 N-86.063 E | (140K) | PQ765947 |  |  |  |  |  |
| 53 | *Armigeres subalbatus* | 27.623 N-86.073 E | (159K) | PQ765948 |  |  |  |  |  |
| 54 | *Armigeres subalbatus* | 27.657 N-86.056 E | (356K) | PQ765949 |  |  |  |  |  |
| 55 | *Armigeres subalbatus* | 27.687 N-86.024 E | (380K) | PQ765950 |  |  |  |  |  |
| 56 | *Collessius pseudotaeniatus* | 26.989 N-85.897 E | CDZMTU-DIPCUL037 | PQ764599 | 8 | 0.84±0.05 | 0.011 | 1.51 | >0.1 |
| 57 | *Collessius pseudotaeniatus* | 27.548 N-86.085 E | CDZMTU-DIPCUL033 | PQ764600 |  |  |  |  |  |
| 58 | *Collessius pseudotaeniatus* | 27.248 N-85.935 E | CDZMTU-DIPCUL038 | PQ764601 |  |  |  |  |  |
| 59 | *Collessius pseudotaeniatus* | 27.548 N-86.085 E | CDZMTU-DIPCUL034 | PQ764602 |  |  |  |  |  |
| 60 | *Collessius pseudotaeniatus* | 27.657 N-86.056 E | CDZMTU-DIPCUL036 | PQ764603 |  |  |  |  |  |
| 61 | *Collessius pseudotaeniatus* | 27.396 N-86.063 E | CDZMTU-DIPCUL035 | PQ764604 |  |  |  |  |  |
| 62 | *Collessius pseudotaeniatus* | 27.396 N-86.063 E | CDZMTU-DIPCUL039 | PQ764605 |  |  |  |  |  |
| 63 | *Collessius pseudotaeniatus* | 27.548 N-86.085 E | CDZMTU-DIPCUL040 | PQ764606 |  |  |  |  |  |
| 64 | *Collessius pseudotaeniatus* | 27.103 N-85.966 E | (143K) | PQ765951 |  |  |  |  |  |
| 65 | *Collessius pseudotaeniatus* | 27.103 N-85.966 E | (153K) | PQ765952 |  |  |  |  |  |
| 66 | *Collessius pseudotaeniatus* | 26.989 N-85.897 E | (373K) | PQ765953 |  |  |  |  |  |
| 67 | *Collessius pseudotaeniatus* | 26.989 N-85.897 E | (378K) | PQ765954 |  |  |  |  |  |
| 68 | *Collessius pseudotaeniatus* | 27.548 N-86.085 E | (R11) | PQ765955 |  |  |  |  |  |
| 69 | *Collessius pseudotaeniatus* | 27.623 N-86.073 E | (R32) | PQ765956 |  |  |  |  |  |
| 70 | *Collessius pseudotaeniatus* | 27.103 N-85.966 E | (R23) | PQ765957 |  |  |  |  |  |
| 71 | *Collessius pseudotaeniatus* | 27.248 N-85.935 E | (359K) | PQ765958 |  |  |  |  |  |
| 72 | *Collessius pseudotaeniatus* | 27.248 N-85.935 E | (368K) | PQ765959 |  |  |  |  |  |
| 73 | *Collessius pseudotaeniatus* | 27.324 N-85.995 E | (379K) | PQ765960 |  |  |  |  |  |
| 74 | *Collessius pseudotaeniatus* | 27.687 N-86.024 E | (R127) | PQ765961 |  |  |  |  |  |
| 75 | *Collessius pseudotaeniatus* | 27.324 N-85.995 E | (R129) | PQ765962 |  |  |  |  |  |
| 76 | *Collessius pseudotaeniatus* | 27.623 N-86.073 E | (R136) | PQ765963 |  |  |  |  |  |
| 77 | *Collessius pseudotaeniatus* | 27.657 N-86.056 E | (R137) | PQ765964 |  |  |  |  |  |
| 78 | *Collessius pseudotaeniatus* | 27.687 N-86.024 E | (R140) | PQ765965 |  |  |  |  |  |
| 79 | *Culex bitaeniorhynchus* | 26.989 N-85.897 E | CDZMTU-DIPCUL041 | PQ764607 | 1 | 0.00±0.00 | 0 | n/a | n.a |
| 80 | *Culex bitaeniorhynchus* | 27.324 N-85.995 E | (R138) | PQ765966 |  |  |  |  |  |
| 81 | *Culex pipiens* | 27.657 N-86.056 E | CDZMTU-DIPCUL042 | PQ764608 | 1 | 0.00±0.00 | 0 | n/a | n.a |
| 82 | *Culex pipiens* | 26.610 N-85.850 E | (152K) | PQ765967 |  |  |  |  |  |
| 83 | *Culex pipiens* | 26.718 N-85.908 E | (155K) | PQ765968 |  |  |  |  |  |
| 84 | *Culex pipiens* | 26.989 N-85.897 E | (156K) | PQ765969 |  |  |  |  |  |
| 85 | *Culex pipiens* | 27.248 N-85.935 E | (158K) | PQ765970 |  |  |  |  |  |
| 86 | *Culex pipiens* | 27.324 N-85.995 E | (161K) | PQ765971 |  |  |  |  |  |
| 87 | *Culex pipiens* | 27.396 N-86.063 E | (R66) | PQ765972 |  |  |  |  |  |
| 88 | *Culex pipiens* | 27.687 N-86.024 E | (146K) | PQ765973 |  |  |  |  |  |
| 89 | *Culex quinquefasciatus* | 27.396 N-86.063 E | CDZMTU-DIPCUL043 | PQ764609 | 1 | 0.00±0.00 | 0 | n/a | n.a |
| 90 | *Culex sasai* | 27.748 N-86.032 E | CDZMTU-DIPCUL044 | PQ764610 | 1 | 0.00±0.00 | 0 | n/a | n.a |
| 91 | *Culex sasai* | 27.721 N-86.019 E | (363K) | PQ765974 |  |  |  |  |  |
| 92 | *Culex sasai* | 27.757 N-86.034 E | (R19) | PQ765975 |  |  |  |  |  |
| 93 | *Gilesius pulchriventer* | 27.748 N-86.032 E | CDZMTU-DIPCUL045 | PQ764611 | 3 | 0.68±0.12 | 0.007 | 2.03 | <0.05 |
| 94 | *Gilesius pulchriventer* | 27.757 N-86.034 E | CDZMTU-DIPCUL047 | PQ764612 |  |  |  |  |  |
| 95 | *Gilesius pulchriventer* | 27.748 N-86.032 E | CDZMTU-DIPCUL046 | PQ764613 |  |  |  |  |  |
| 96 | *Gilesius pulchriventer* | 27.748 N-86.032 E | (R6) | PQ765976 |  |  |  |  |  |
| 97 | *Gilesius pulchriventer* | 27.748 N-86.032 E | (R131) | PQ765977 |  |  |  |  |  |
| 98 | *Gilesius pulchriventer* | 27.748 N-86.032 E | (R139) | PQ765978 |  |  |  |  |  |
| 99 | *Gilesius pulchriventer* | 27.748 N-86.032 E | (R128) | PQ765979 |  |  |  |  |  |
| 100 | *Gilesius pulchriventer* | 27.748 N-86.032 E | (137K) | PQ765980 |  |  |  |  |  |
| 101 | *Lutzia halifaxii* | 27.324 N-85.995 E | CDZMTU-DIPCUL048 | PQ764614 | 1 | 0.00±0.00 | 0 | n/a | n.a |
| 102 | *Lutzia halifaxii* | 26.865 N-85.963 E | (R29) | PQ765981 |  |  |  |  |  |
| 103 | *Lutzia halifaxii* | 26.989 N-85.897 E | (R108) | PQ765982 |  |  |  |  |  |
| 104 | *Lutzia halifaxii* | 27.396 N-86.063 E | (R63) | PQ765983 |  |  |  |  |  |
| 105 | *Toxorhynchites splendens* | 27.324 N-85.995 E | CDZMTU-DIPCUL049 | PQ764615 | 1 | 0.00±0.00 | 0 | n/a | n.a |
| 106 | *Toxorhynchites splendens* | 27.103 N-85.966 E | (R3) | PQ765984 |  |  |  |  |  |
| 107 | *Toxorhynchites splendens* | 27.396 N-86.063 E | (R12) | PQ765985 |  |  |  |  |  |
| 108 | *Toxorhynchites splendens* | 27.623 N-86.073 E | (R13) | PQ765986 |  |  |  |  |  |
| 109 | *Toxorhynchites splendens* | 27.103 N-85.966 E | (R14) | PQ765987 |  |  |  |  |  |
| 110 | *Toxorhynchites splendens* | 27.324 N-85.995 E | (R15) | PQ765988 |  |  |  |  |  |
| 111 | *Toxorhynchites splendens* | 27.396 N-86.063 E | (R16) | PQ765989 |  |  |  |  |  |
| 112 | *Toxorhynchites splendens* | 27.623 N-86.073 E | (R17) | PQ765990 |  |  |  |  |  |
| 113 | *Toxorhynchites splendens* | 27.396 N-86.063 E | (R28) | PQ765991 |  |  |  |  |  |
| 114 | *Toxorhynchites splendens* | 27.623 N-86.073 E | (166K) | PQ765992 |  |  |  |  |  |
